# Drug-tolerant persister cells in acute myeloid leukemia: pressing challenge and promising new strategies for treatment

**DOI:** 10.3389/fmed.2025.1586552

**Published:** 2025-05-14

**Authors:** Meng Li, Xiaoli Wang, Wenjuan He, Hao Zhou

**Affiliations:** ^1^Institute of Hematology, Union Hospital, Tongji Medical College, Huazhong University of Science and Technology, Wuhan, China; ^2^Department of Obstetrics and Gynecology, Union Hospital, Tongji Medical College, Huazhong University of Science and Technology, Wuhan, China

**Keywords:** drug tolerant persister, acute myeloid leukemia, chemotherapy resistance, metabolic remodelling, relapse, minimal residual disease

## Abstract

Acute myeloid leukemia (AML) exhibits a pronounced ability to develop drug resistance and undergo disease relapse. Recent research has noticed that resistance to treatments could substantially be attributed to drug-tolerant persister (DTP) cells, which are capable of surviving under therapeutic pressures. These are transient, reversibly dormant cells with the capability to act as a reservoir for disease relapse. DTP cells utilize diverse adaptive strategies to optimize the ecological niche, undergo metabolic reprogramming, and interact with microenvironment. The persister state of AML is established through transient cellular reprogramming, thus allowing cells to survive the initial phase of drug therapy and develop drug resistance. Our review explores the identification and phenotypic characteristics of AML DTP cells, as well as their clinical relevance. We summarize the mechanisms underlying the persistence of AML DTP cells and the molecular attributes that define the DTP state. We further address the current challenges and future prospects of DTP-targeting approaches. Understanding these features may provide critical insights into novel therapeutic strategies aimed at targeting AML DTP cells, especially in the new era of immunotherapy against AML.

## 1 Introduction

Cancer continues to pose an important global public health challenge (1, 2). Acute myeloid leukemia (AML) is a malignant hematologic tumor originating from myeloid hematopoietic stem cells, characterized by the excessive proliferation of abnormal leukemic cells in the bone marrow, which impairs normal hematopoiesis and leads to clinical manifestations such as anemia, bleeding, and infections (3–5). AML is the most prevalent form of acute leukemia in adults, with its incidence significantly increasing with age, particularly in individuals over 60 years old (6). Acute myeloid leukemia is more frequent in adults but is also common in children, with higher incidence rates in higher Human Development Index (HDI) settings (1, 7). Clinical features include abnormal peripheral blood leukocyte counts, anemia, thrombocytopenia, and extensive infiltration of the bone marrow by immature myeloid cells (3, 8). The standard treatment for AML primarily involves intensive chemotherapy, targeted therapy, immunotherapy and hematopoietic stem cell transplantation (HSCT) (8–12). On the one hand, while drug can induce remission to some extent, drug resistance and relapse remain significant challenges in the treatment of AML. On the other hand, while HSCT can effect a radical cure, complication challenges include graft failure, graft-versus-host disease (GVHD), and relapse could not be avoided (13, 14). Chemotherapy, the traditional treatment for AML, utilizes cytotoxic drugs to kill rapidly dividing leukemic cells. However, many patients develop resistance after initial remission, resulting in disease relapse (10). In recent decades, targeted therapies and immunotherapies have provided new hope for AML treatment. Targeted therapy inhibits the growth of leukemic cells by targeting specific genetic mutations or signaling pathways, while immunotherapy functions by stimulating the individual’s immune system to assault leukemic cells (8, 9). Although these novel therapies have demonstrated remarkable efficacy in selected patients, the complexity of resistance mechanisms remains a formidable challenge. The complexity of resistance mechanisms is one of the main factors that limits the effectiveness of AML treatment. Resistance can be crudely divided into two forms-genetic and non-genetic. Genetic resistances are in general caused by genetic mutations which may alter targets of drugs or activate alternative signaling pathways. However, non-genetic resistance mechanisms are also crucial, particularly the formation of drug-tolerant persister (DTP) cells (13, 15, 16).

DTP cells are a subset of cells that survive drug treatment and maintain a low proliferative state. DTP cells were initially derived from bacterial resistance studies, describing a non-genetic resistance phenomenon in which certain bacteria can survive and repopulate during antibiotic treatment (17–19). In oncology, DTP cells are defined as a cell subset that can endure the toxic effects of drugs and remain viable under drug treatment, with non-genetic, reversible resistance (20–23). Key characteristics of DTP cells include non-genetic resistance, reversibility, phenotypic plasticity, and metabolic remodeling (23–27). These cells can enter a dormant state under drug pressure, evading drug-induced cell death, and can resume proliferation once the drug is removed (20, 22, 23). In recent decades, substantial progress has been achieved in studying DTP cells in solid tumors, with evidence of their widespread presence in lung cancer, breast cancer, gastrointestinal tumors, and other types (22, 28–32). DTP cells can survive and maintain a low proliferative state under drug pressure through epigenetic reprogramming, metabolic remodeling, and intercellular signaling. It has been proved that DTP cells also exist in AML and play a critical role in drug resistance and recurrence (13, 15, 33). Thus, the research on DTP cells in AML is significant for understanding the resistance mechanism in AML and will provide a theoretical basis for finding new therapeutic strategies.

## 2 DTP cells in AML: characteristics and phenotypes

### 2.1 Identification and phenotypic characteristics of DTP cells in AML

#### 2.1.1 Methods for identifying DTP cells during experiments

Identifying DTP cells is crucial for elucidating their resistance mechanisms in AML. During experiments, DTP cells are primarily identified through *in vitro* experiments, single-cell sequencing, and flow cytometry.

*In vitro* experiments expose AML cells to chemotherapeutic drugs for prolonged periods of time to simulate clinical chemotherapy conditions, thereby assessing cell survival and proliferation. For instance, Morgenstern et al. treated AML cell lines (including MV4-11, THP-1 and MOLM-13) with high concentrations of Ara-C and daunorubicin, collected the surviving cells and analyzed their phenotypic characteristics via flow cytometry, successfully establishing a DTP cell model (34). The results showed that DTP cells exhibited significant survival capabilities and slow proliferation after chemotherapy, which is closely associated with chemotherapeutic tolerance.

Flow cytometry, through specific cell surface marker staining combined with fluorescence labeling and cell sorting techniques, detects changes in the cell cycle of DTP cells and the expression of drug resistance-related markers (35, 36). For example, CD44 and MDR1 are cell surface markers associated with drug resistance (37). By using flow cytometry to detect the expression levels of these markers in DTP cells, researchers can screen out DTP cells with drug resistance. This technique identifies DTP cells and helps researchers further understand their drug resistance mechanisms.

Single-cell sequencing technology can deeply analyze the gene expression profiles of individual DTP cells, revealing cellular heterogeneity (38–40). For example, Gebru et al. revealed the heterogeneity of DTP cells at the gene expression and transcriptome levels (33). After treatment, DTP cells exhibited unique transcriptomic features significantly different from those of normal hematopoietic stem cells (HSCs) and leukemia stem cells (LSCs). This difference not only helps researchers further understand the drug resistance mechanisms of DTP cells but also provides an important molecular basis for developing targeted therapeutic strategies. Although Single-cell sequencing technology is effective in identifying DTP cells, few patients can afford it in clinical applications.

In summary, by combining *in vitro* experiments, flow cytometry, and single-cell sequencing technology, researchers can comprehensively identify DTP cells and deeply understand their drug resistance mechanisms. These technical means provide important tools and theoretical support for developing new therapeutic strategies (41) (Table 1).

**TABLE 1:** Characteristics of DTP cells across three recent studies in AML.

Characteristics	van Gils et al. (13)	Gebru et al. (33)	Morgenstern et al. (15)
Induction method	Treatment with doxorubicin to induce drug-tolerant clones in K562 cells.	Treatment with FLT3 inhibitors (quizartinib) in FLT3-ITD mutant AML cell lines to induce DTPs.	Treatment with combination chemotherapy (Daunorubicin and Ara-C) in AML cell lines and primary AML patient samples to induce DTP cells.
Definition of DTP cells	Drug-tolerant leukemia cells that survive chemotherapy, characterized by stem cell features and lack of H3K27me3 or H3K4me3 upregulation.	Drug-tolerant leukemia cells that survive FLT3 inhibitor treatment, characterized by upregulation of inflammatory pathways.	Drug-tolerant leukemia cells that survive chemotherapy, characterized by increased plasma membrane rigidity.
Major features	Cell cycle arrest (G1 or G2-M phase); stem cell features with high expression of CD44 and MDR1; lack of H3K27me3 or H3K4me3 upregulation; reversible (regain sensitivity upon drug removal)	Cell cycle arrest (G1 or G2-M phase); upregulation of inflammatory pathway genes; reversible (regain sensitivity upon drug removal); increased sensitivity to glucocorticoids	Cell cycle arrest (G1 or G2-M phase); increased plasma membrane rigidity; reversible (regain sensitivity upon drug removal); increased sensitivity to immune cell killing
Recovery of drug sensitivity (time)	Approximately 10 weeks	Approximately 28 days	Approximately 14 days
Recovery of drug sensitivity (condition)	Cultured in drug-free medium	After drug removal	After chemotherapy removal

This table provides a structured comparison of the methods used to induce DTP cells, their definitions, key characteristics, and the conditions under which they regain drug sensitivity across the three studies.

#### 2.1.2 Phenotypic characteristics of DTP cells

HSCs are a small pool of pluripotent cells, which are origin of all blood lineages (42). LSCs are resistant cells with long-term self-renewal capacity that drive clonal outgrowth (43). Phenotypically, DTP cells overlap with but differ from normal HSCs and LSCs (44–47). In terms of proliferation, HSCs have self-renewal and differentiation capabilities, capable of differentiating into various blood cells; LSCs have self-renewal and drug resistance but abnormal differentiation, and can initiate and maintain leukemia development; DTP cells are a subpopulation of drug-resistant cells formed under therapeutic stress, exhibiting higher drug resistance and lower proliferation rates (34). After chemotherapeutic treatment, DTP cells commonly undergo cell cycle arrest at the G1 or G2-M phase, which contributes to their transient evasion of drug-induced cytotoxicity. In contrast, HSCs and LSCs tend to remain in a quiescent state, though through distinct mechanisms. Most HSCs reside in a reversible G0 phase under steady-state conditions. While LSCs may also pause at certain stages of the cell cycle, they can re-enter the cycle once drug resistance develops, thereby contributing to disease persistence or relapse. DTP cells may highly express drug resistance-related proteins (such as MDR1) and upregulate CD36 expression regarding cell surface markers (39). In contrast, LSCs aberrantly express CD9, CD25, CD69, CD93, CD96, CD371/CLL-1, IL-1RAP and typically lack CD26 and CD90. Higher expression of CD33 and CD123 compared to HSCs (48). In terms of metabolic activity, after treatment with chemotherapeutic drugs, DTP cells enter a low proliferative state, allowing them to survive under drug stress (20, 39).

Regarding cell morphology, DTP cells exhibit increased membrane rigidity after treatment, which may be related to chemotherapeutic resistance but also increase their sensitivity to T cell-mediated killing (34). Although DTP cells may acquire “stemness” characteristics similar to LSCs through epigenetic and transcriptomic reprogramming, thereby enhancing drug resistance, once the therapeutic stress is removed, DTP cells can regain drug sensitivity. This indicates that the drug resistance of DTP cells is a dynamic, non-genetic adaptive change (49) (Table 2).

**TABLE 2:** Comparison of the characteristics of normal hematopoietic stem cells, leukemia stem cells, and DTP cells.

Characteristics	Normal hematopoietic stem cells (HSCs)	Leukemia stem cells (LSCs)	DTP cells
Phenotype	Capable of self-renewal and differentiation into various blood cells	Possess self-renewal and drug resistance but exhibit abnormal differentiation abilities and can initiate and sustain leukemia development	A subpopulation of drug-resistant cells formed under treatment stress, exhibiting higher drug resistance and lower proliferation rates
Proliferation ability	Capable of self-renewal and differentiation into various blood cells	Possess self-renewal and drug resistance but exhibit abnormal differentiation abilities	Exhibit higher drug resistance and lower proliferation rates
Cell cycle	Able to progress normally through the cell cycle	Able to progress normally through the cell cycle, though they may stall at certain stages	After chemotherapy drug treatment, the cell cycle stalls in the G1 or G2-M phase
Cell surface markers	Express CD43, CD44, CD90, CD105, CD114, CD117, CD133, CD135, and ROBO4. Do not express CD26, CD96, CD243, CD271, CD309, CLL-1, or IL-1RAP.	Aberrantly express CD9, CD25, CD69, CD93, CD96, CD371/CLL-1, and IL-1RAP. Higher expression of CD33 and CD123 compared to HSCs. Typically lack CD26 and CD90.	May highly express drug resistance-related proteins (e.g., MDR1). Can acquire LSC-like markers (e.g., CD25, CD33, CD123) under treatment stress.
Metabolic activity	Maintain higher metabolic activity to support normal hematopoietic function	Maintain higher metabolic activity to support leukemia development	Enter a low proliferative state after chemotherapy drug treatment to survive under drug pressure
Cell morphology	Normal cell morphology with higher membrane flexibility	Abnormal cell morphology with potentially increased membrane flexibility	Exhibit higher membrane rigidity after treatment, which may be associated with chemotherapy resistance
Drug resistance	Sensitive to chemotherapy drugs	Possess drug resistance and can resist chemotherapy drugs	Possess higher drug resistance, but can regain drug sensitivity once treatment pressure is removed
Adaptive changes	No significant adaptive changes	Acquire drug resistance through genetic and epigenetic alterations, enabling them to resist treatment	Acquire “stemness” similar to LSCs through epigenetic and transcriptomic reprogramming to enhance drug resistance

### 2.2 Clinical relevance of DTP cells in AML

#### 2.2.1 Chemotherapy and targeted therapy resistance

DTP cells are closely related to the treatment response of AML and are one of the key factors leading to resistance to chemotherapy and targeted therapy. Many AML patients show a good initial response to treatment but often face the dilemma of recurrence (50). One important reason is that DTP cells can survive under the action of chemotherapeutic drugs and enter a low proliferation state, just like embryos in diapause, thereby evading drug killing (39, 51, 52). Morgenstern et al. conducted studies revealed a unique survival mechanism of DTP cells that when acutely exposed to daunorubicin and Ara-C, a subpopulation of AML cells would transiently augment membrane rigidity to evade destruction. However, after the chemotherapeutic drugs were removed, the membrane hardness would return to baseline levels, and the cells would regain sensitivity to chemotherapy and restore proliferation (15). Furthermore, DTP cells also exhibit resistance to targeted therapy. In FLT3-mutant AML, after treatment of FLT3 inhibitors, the inflammatory pathway is upregulated in DTP cells, leading to drug resistance (33). AML cells become resistant to venetoclax combined with azacitidine (ven/aza) therapy by increasing nicotinamide metabolism and thus driving OXPHOS (53). The complexity of DTP cells drug resistance mechanisms in AML indicates that a combination of multiple therapeutic strategies is needed. Both their intrinsic survival mechanisms and their interactions with the ecological niche need to be addressed.

#### 2.2.2 Transition from minimal residual disease to relapse

DTP cells are key in transitioning from minimal residual disease (MRD) to relapse in AML. After treatment, DTP cells, as an important component of MRD, persist and remain quiescent (50, 54). However, after removal of therapeutic stress, DTP cells can re-enter the cell cycle and begin to proliferate rapidly. This reactivation often marks the beginning of relapse. Studies have shown that DTP cells can resist the clearance by immune cells in the bone marrow microenvironment through immune evasion and other mechanism, gradually restore proliferative capacity, and thereby trigger disease relapse (55–57). The transition from MRD to relapse is driven by the resilience and adaptability of DTP cells. DTP cells maintain their drug tolerance under chemotherapeutic stress and cause relapse through non-genetic resistance mechanisms, metabolic remodeling, cellular signaling modification, and the effective of leukemic microenvironment, etc. These pose a serious challenge for the complete cure of AML.

## 3 Drug resistance mechanisms of DTP cell

### 3.1 Non-genetic resistance mechanisms

#### 3.1.1 Epigenetic reprogramming

Epigenetic reprogramming is crucial in forming DTP cells, with histone modifications and chromatin remodeling being core factors. DTP cells maintain their drug-resistant phenotype through dynamic epigenetic modifications. For instance, van Gils et al. found that anthracycline-induced AML DTP cells exhibited a hypomethylated state of H3K27me3 and H3K4me3, accompanied by increased expression of the KDM6 demethylase and downregulation of EZH1/2 methyltransferases (13). This epigenetic reprogramming activated stem cell-related genes (such as CD44 and MDR1), endowing DTP cells with leukemia stem cell-like characteristics. Targeted inhibition of KDM6 can restore H3K27me3 levels, inducing DTP cell apoptosis by upregulating pro-apoptotic proteins STAT5B and BCL2 (13). Preclinical models have shown that the KDM6 inhibitor GSK-J4 significantly reduces the burden of AML minimal residual disease, highlighting the potential of epigenetic regulators in eliminating DTP cells (58, 59).

#### 3.1.2 Transcriptome reprogramming

In DTP cells, transcriptome reprogramming is one of the mechanisms of non-genetic resistance. This process involves activation and repression of key transcription factors and gene regulatory networks. For example, upregulation of inflammatory pathways such as the IL-6/STAT3 axis plays an important role in the survival of DTP cells (60). Activation of STAT3, a activator of transcription responsive to inflammatory signals, promotes cell survival and expression of drug efflux genes (61). In addition, activation of stem cell-associated genes such as CD44 and MDR1 confers greater plasticity and drug resistance to DTP cells (62). CD44 is a marker commonly associated with stemness that contributes to the cell’s ability to adapt to different microenvironmental conditions, while MDR1 is a drug efflux transporter (63, 64). These reprogramming collectively allow DTP cells to withstand drug stress.

#### 3.1.3 Translational reprogramming

**FIGURE 1:**
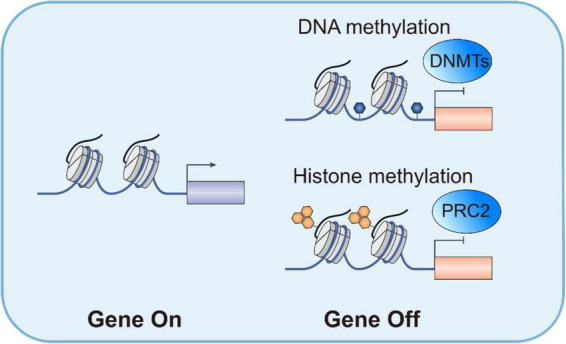
Epigenetic and transcriptional regulation.

The emergency of DTP cells is closely related to translational reprogramming, particularly the role of translation regulatory factors such as eIF4A (65). Changes in these factors directly affect protein synthesis, thereby influencing cellular drug resistance. Studies have shown that DTP cells exhibit significant changes in mRNA translation efficiency after chemotherapy, with eIF4A particularly critical (66). eIF4A, an RNA helicase, alters its activity to affect mRNA translation efficiency and protein synthesis (67). Under stress conditions, changes in eIF4A activity prioritize the translation of proteins related to drug resistance and cell survival, thereby enhancing cellular drug resistance. Inhibition of eIF4A can significantly reduce the survival capacity of DTP cells (34) (Figure 1).

### 3.2 Metabolic remodeling

The DTP cells exhibit unique metabolic characteristics. These cells either predominantly utilize oxidative phosphorylation (OXPHOS) or exhibit a hybrid metabolic phenotype that relies on both OXPHOS and the pentose phosphate pathway of glycolysis (68). This metabolic remodeling helps DTP cells maintain energy supply and redox balance under therapeutic stress.

DTP cells enhance the generation of reactive oxygen species (ROS) production by upregulating mitochondrial function, activating redox signaling pathways and promoting cell survival (68–70). In DTP cells, ROS are involved in oxidative damage and homeostatic processes such as metabolism, immune response, cell growth, and differentiation. For example, DTP cells enhance OXPHOS via modulating the histone H3K4 demethylase KDM5B, exhibiting a quiescent phenotype characterized by increased oxygen consumption and elevated levels of hydrogen peroxide. Study reveals that mitochondrial functions, particularly high OXPHOS status, contribute to cytarabine-resistance in AML (71).

**FIGURE 2:**
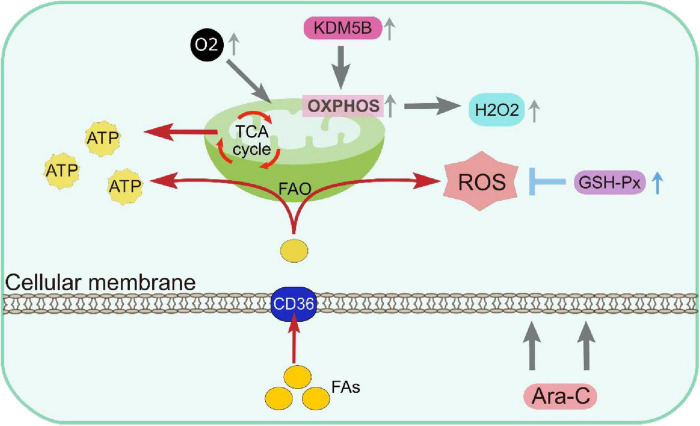
Metabolic remodeling resulting in DTP cells depending on mitochondrial respiration for energy production and having increased antioxidant capacity. ATP, Adenosine triphosphate; FAO, fatty acid β-oxidation; GSH-Px, glutathione peroxidases; OXPHOS, oxidative phosphorylation; ROS, reactive oxygen species.

Furthermore, DTP cells combat oxidative stress by upregulating glutathione peroxidases (GSH-Px), maintaining redox balance (72). GSH-Px protect DTP cells from oxidative damage by catalyzing the detoxification of ROS and preventing lipid peroxidation-induced membrane damage. Based on it, on the one hand, DTP cells maintain metabolic activity and prevent lipotoxicity by enhancing fatty acid oxidation (FAO) which further enhancing the survival capacity of DTP cells (73, 74). On the other hand, DTP cells showed an enhanced absorption of fatty acids and exhibited higher levels of elongated fatty acids, triglycerides, and sphingomyelins. Alterations in lipidome composition result in stiffer plasma membranes and play a role in chemoresistance (15) (Figure 2).

### 3.3 Cellular signaling modification

**FIGURE 3:**
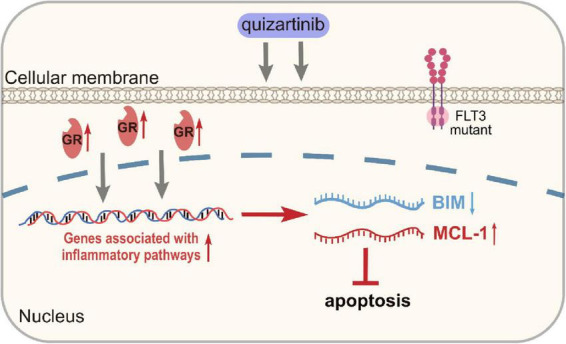
Up-regulation of genes associated with inflammatory pathways in DTP cells. GR, glucocorticoid receptors.

In AML, the underlying mechanisms of the emergence of DTP cells involve various signaling pathways. These cells show upregulated prosurvival signaling pathways, including the MAPK and PI3K-AKT pathways, which promote their survival and propagation (75). In FLT3-mutant AML, treatment with FLT3 inhibitors like quizartinib mediates the augmentation of inflammatory pathways in DTP cells, characterized by the activation of transcription factors and cytokines involved in inflammation (33). Anti-inflammatory glucocorticoids can target this inflammatory response, which enhances cell death by increasing the proapoptotic protein BIM and decreasing the antiapoptotic protein MCL-1. These signaling modifications highlight DTP cells’ dynamic and adaptive nature, providing multiple targets for therapeutic intervention to overcome drug resistance in AML (76) (Figure 3).

### 3.4 Leukemic microenvironment

Mounting evidence suggest that cancer-associated fibroblasts (CAFs) represent the predominant fraction of stromal cells inside the solid tumor microenvironment (77). While in the case of AML, the development of DTP cells is intricately linked to cell-cell interactions inside the leukemic microenvironment (78). Within the leukemia microenvironment, bone marrow stromal cells may display CAF characteristics, which secret multiple cytokines to enhance leukemia proliferation, infiltration, and niche modification (79, 80). Malignant leukemia-associated fibroblasts (LAFs) might alter the bone marrow microenvironment, creating a more hostile and chemoresistant niche (81). While the precise functions and mechanisms of LAFs are not fully understood, it is evident that LAFs in bone marrow may be associated with accelerated leukemia growth and increased treatment resistance. Certain chemotherapeutic agents, including cytarabine and anthracycline, have been observed to potentially stimulate the formation of LAFs (81–83). Generally, LAFs may exhibit unique characteristics and are instrumental in therapeutic resistance.

**FIGURE 4:**
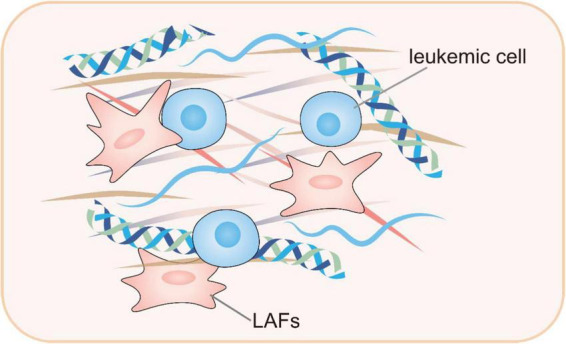
Leukemic-microenvironment interaction, particularly involving leukemia-associated fibroblasts within the reactive stroma. LAFs, leukemia-associated fibroblasts.

In addition to LAFs, the leukemic microenvironment also includes other stromal cells that support the survival of DTP cells by secreting cytokines and chemokines and offering physical support (84, 85). After engulfing apoptotic cell debris, macrophages release anti-apoptotic signals and secrete inflammatory factors such as IL-6 and TNF-α, which activate survival signaling pathways in DTP cells and promote their survival (86). The formation of DTP cells is also substantially influenced by hypoxia and nutrient deprivation, which are also microenvironmental factors. Intracellular hypoxia-inducible factors (HIF-1α) are activated in hypoxic conditions, thereby initiating the HIF pathway (29, 87). This pathway initiates metabolic remodeling in DTP cells, thereby improving their adaptability to hypoxic environments and upregulating drug resistance-related genes. Under nutritional scarcity, DTP cells modify their metabolic processes by initiating autophagy and metabolic modification so they adjust to the hostile environment and ensure survival (88, 89). Research indicates that DTP cells, when deprived of resources, markedly increase the oxidization of fatty acids and OXPHOS process, effectively using scarce resources to sustain the utilization of energy and ensuring cellular survival (90) (Figure 4).

### 3.5 Immune evasion mechanisms

DTP cells evade the recognition and elimination by the immune system via multiple means, presenting a significant and emerging challenge in treating AML, especially in the new era of immunotherapy against AML (91, 92). One of DTP cells’ important immune evasion strategies is the upregulation of immune checkpoint molecules (93). For instance, the increased expression of PD-L1 and CTLA-4 can suppress immune cells’ activity, hinder T cells’ killing function, and make it difficult for them to effectively recognize and eliminate DTP cells (94, 95). In addition, DTP cells are capable of secreting immunosuppressive factors, including TGF-β and IL-10, which collectively create an immunosuppressive microenvironment, attracting the aggregation of immunosuppressive cells including myeloid-derived suppressor cells (MDSCs) and regulatory T cells (96–100). These immunosuppressive cells further suppress immune responses, protecting DTP cells, enabling them to escape immune system attacks, persist in the body, and cause disease recurrence.

### 3.6 The differences resistance mechanisms between DTP cells and LSCs

Drug resistance in DTP cells is more related to non-genetic factors and is a dynamic adaptive change induced in the cells under drug stress, which is reversible (101). Drug resistance in LSCs is a naturally occurring trait and is more dependent on their intrinsic biological properties which endow them with stronger genetic and epigenetic stability (43). Here’s a comparison of the different angles.

#### 3.6.1 Epigenetic regulation

DTP cells induce reprogramming of gene expression through activation of histone demethylases such as KDM5A, and epigenetic changes such as DNA methylation and acetylation, resulting in tolerance to drugs (13). LSCs are more dependent on mutations in genes such as DNMT3A and TET2, which affect the methylation status of DNA and thus modulate the gene expression program, allowing LSCs to acquire drug resistance (102).

#### 3.6.2 Cellular signaling modification

The formation of DTP cells and drug resistance are closely related to the alteration of various signaling pathways, such as EGFR, JAK/STAT, PI3K/AKT, MAPK and other signaling pathways (103, 104). Abnormal activation or inhibition of these pathways affects cell proliferation, survival and response to drugs. A common mechanism of drug resistance in LSCs involves the activation of downstream signaling pathways by mutations in FLT3, IDH1/2, and other genes (105, 106).

#### 3.6.3 Leukemic microenvironment

DTP cells are induced by drugs to alter the tumor microenvironment through processes such as epithelial-mesenchymal transition (EMT), which enables the cells to better adapt to drug stress and enhance tolerance to drugs (107). For example, the EMT process allows cells to acquire migratory and invasive capabilities, while altering their interactions with the extracellular matrix, thereby affecting drug uptake and sensitivity (108). LSCs are usually located in specific regions of the bone marrow microenvironment, such as the hypoxic region (niche), where the microenvironment provides protection for LSCs (109). In the hypoxic environment, the expression of factors such as HIF1a is increased, which reduces sensitivity to chemotherapeutic agents by regulating gene expression to bring LSCs into a resting state (110).

#### 3.6.4 Metabolic remodeling

DTP cells usually change their metabolic mode from aerobic glycolysis to mitochondrial respiration for energy. Increased levels of ROS are accompanied by activation of the antioxidant system, further enhancing drug resistance (68). LSCs dependent on mitochondrial OXPHOS for energy production and utilize primarily amino acids and fatty acids as energy sources (111, 112). LSCs use a variety of molecular and metabolic mechanisms to maintain a ROS-Low state (113), and rely on OXPHOS for the production of high-energy compounds. This unique metabolism allows LSCs to survive in a low-glycemic, low-oxygen microenvironment and develop resistance to chemotherapeutic drugs.

#### 3.6.5 Redox regulation

DTP cells survive drug-induced oxidative stress by increasing the expression of enzymes such as glutathione peroxidase 4 (GPX4) and aldehyde dehydrogenase (ALDH), scavenging harmful substances generated by oxidative stress, and maintaining intracellular redox balance (72, 114). LSCs maintain low levels of ROS through multiple mechanisms to avoid cellular damage from oxidative stress (115, 116). For example, LSCs can scavenge ROS by increasing glutathione production, activating FOXO transcription factors (117), and mitochondrial autophagy to maintain cellular stability and function (118).

In summary, these differences provide different targets and ideas for therapeutic strategies against DTP cells and LSCs.

## 4 Potential therapeutic strategies for DTP cells in AML

### 4.1 Targeting metabolic pathways in DTP cells

DTP cells exhibit remarkable metabolic adaptability, with significantly enhanced OXPHOS and fatty acid oxidation activity (68). Therefore, it is considered a promising therapeutic strategy for inhibiting these metabolic pathways. Studies have shown that GPX4 inhibitors can disrupt the antioxidant defense system of DTP cells, accumulating intracellular reactive oxygen species and ultimately causing cell death (72). ACOX1 inhibitors can suppress fatty acid oxidation, reducing the energy supply of cells and thereby weakening the survival capacity of DTP cells (119). Although still some way from clinical translation, theoretically, these inhibitors used alone or in combination, may selectively kill DTP cells.

In addition to single-target inhibitors, the therapeutic strategy of simultaneously targeting multiple metabolic pathways also holds potential efficacy. For example, drugs that inhibit both OXPHOS and fatty acid oxidation can block multiple energy metabolism pathways of DTP cells, thereby enhancing their sensitivity to treatment (69). On this basis, combining drugs that inhibit OXPHOS and fatty acid oxidation with other targeted drugs (such as inhibitors of specific signaling pathways) can interfere with the metabolic processes and survival signals of DTP cells from multiple dimensions (74). For instance, the combination of GPX4 inhibitors and FLT3 inhibitors can, on the one hand, disrupt the antioxidant capacity of DTP cells, resulting in an abundance of intracellular ROS and causing cell damage (120); on the other hand, it can block abnormal signal transduction, inhibiting cell proliferation and survival, thereby synergistically inhibiting the growth of DTP cells (28, 33). Moreover, combining metabolic inhibitors with immunotherapeutic drugs (such as CAR-T cell therapy or immune checkpoint inhibitors) is expected further to enhance the therapeutic effect (24, 121–125). Immunotherapeutic drugs can activate the body’s immune system, enhancing the immune cells’ ability to recognize and kill DTP cells (126). Combined with metabolic inhibitors, they can act on DTP cells from both metabolic and immune aspects, bringing new hope for overcoming the drug resistance challenges caused by DTP cells in leukemia.

### 4.2 Targeting epigenetic and transcriptional regulation

In AML, non-genetic mechanisms are used by DTP cells to withstand drug therapy. Therapeutic strategies targeting this could be potential treatments for DTP cells in AML. Some of the findings so far are followed. In AML, targeting the histone demethylase KDM6 by epigenetic reprogramming removes DTP cells, which were found to be characterized by low H3K27me3 and H3K4me3 levels (13). Histone demethylase inhibitors, such as KDM5 and KDM6 inhibitors, can regulate the methylation status of histones, thereby correcting abnormal gene expression patterns (127, 128). The KDM6B inhibitor GSK-J4 significantly augments the sensitivity of DTP cells in AML to chemotherapeutic drugs (129). GSK-J4 increases H3K27me3 levels by reducing the KDM6B enzymatic activity (130), diminishes the expression of drug resistance-related genes in AML (131).

Targeting transcription factors is also an important strategy for treating DTP cells. For example, RXR antagonists and EZH2 inhibitors can influence the gene expression of DTP cells by modulating the activity of transcription factors. RXR antagonists can block the activity of specific transcription factors and interfere with the transcriptional regulatory network of DTP cells, thereby inhibiting the expression of drug resistance-related genes of DTP cells (132). EZH2 inhibitors, on the other hand, can modulate the chromatin state, alter the microenvironment of gene transcription, and thereby inhibit the growth and survival of DTP cells (133). With the continuous development and clinical application of these targeted drugs, breakthroughs are expected in precisely eliminating DTP cells and improving the therapeutic effect of AML.

### 4.3 Aiming at immune evasion mechanisms

At present, immunotherapy for AML is still challenged by issues like immune escape and antigen escape, which restrict its application (134). Therefore, combining immunotherapy with targeted therapy for DTP cells holds promise for providing more effective strategies for AML treatment. Chimeric antigen receptor (CAR) T-cell therapy involves genetically modifying a patient’s own T cells to express a chimeric antigen receptor that can recognize and target specific cancer cells, leading to their destruction (135, 136). For example, CAR-T cell therapy can precisely target surface antigens of DTP cells, achieving precise recognition and killing of DTP cells (137). However, the immune evasion mechanisms of DTP cells often limit the therapeutic effect when CAR-T cell therapy is used alone (138, 139). Research has demonstrated that the concurrent application of immune checkpoint inhibitors including PD-1 inhibitor and CTLA-4 inhibitor can block immune checkpoint signals, relieve the inhibitory effect of DTP cells upon T cells, significantly enhance T cell activity, and consequently enhance the lethality of CAR-T cells against DTP cells (140–143). Moreover, combining immunotherapy with targeted therapy for DTP cells (such as metabolic inhibitors or epigenetic inhibitors) may work synergistically from multiple aspects, more effectively eliminating DTP cells, further improving the overall therapeutic effect of AML.

### 4.4 Blocking the formation and evolution of DTP cells

DTP cells possess high phenotypic plasticity, enabling them to flexibly alter their phenotype and function according to microenvironmental conditions (101, 144, 145).This characteristic allows DTP cells to adapt and evolve various drug resistance mechanisms under therapeutic stress, significantly increasing the difficulty of AML treatment. Therefore, blocking DTP cells’ phenotypic transition and drug resistance evolution has become a key strategy in AML treatment. On one hand, the suppression of key transcription factor activities virtually prevents DTP cells from phenotypic shifting toward more highly drug-resistant cell types (13, 101). On the other hand, various targeted drugs, including epigenetic and metabolic inhibitors, might simultaneously block different mechanisms of DTP cell drug resistance, therefore achieving a far better therapeutic response (33, 68). In addition, interfering with the signaling pathways of DTP cells and inhibiting their transitions between different drug-resistant states can limit the development of their drug resistance (68). It is worth noting that continuously monitoring the phenotype and molecular characteristics of DTP cells in patients and promptly adjusting treatment plans based on this information is crucial for effectively inhibiting the evolution of DTP cells and improving the success rate of AML treatment (101). This comprehensive strategy is expected to bring a better treatment outlook for AML patients, reduce the risk of disease recurrence, and improve patient prognosis.

## 5 Conclusion and expectations in the field of AML DTP

### 5.1 Importance of DTP cell research in AML

DTP cells occupy a central position in the drug resistance mechanisms of AML. Numerous studies have shown that DTP cells can survive under therapeutic stress and gradually develop drug resistance through various complex mechanisms, including epigenetic reprogramming, transcriptome reprogramming, and metabolic remodeling (38, 68, 146–149). During treatment, these cells exhibit temporary proliferation inhibition, but after the therapeutic stress is removed, they can rapidly resume proliferation and regain drug resistance (24, 150). Therefore, DTP cells are a key factor in AML relapse and drug resistance, posing a significant challenge to leukemia treatment. DTP cells’ reversibility and dynamic nature make them a highly promising therapeutic target (39). Since their drug resistance is formed based on non-genetic mechanisms, research on these mechanisms can help develop new therapeutic strategies, providing the possibility to overcome drug resistance.

### 5.2 Promising new research directions

DTP cells are cell cycle arrested and in a lower proliferation rates after being stressed by drugs. Is this arrest beneficial or detrimental to chemotherapy response? On the one hand, the arrest may reflect DNA damage caused by chemotherapeutic agents; on the other hand, it may serve as a protective mechanism by which the cells evade drug-induced killing and gain time for DNA repair. We may be able to block this protective mechanism of DTP cells by synergizing with other drugs. Clinically common, non-oncology drugs may be one of the directions that can be tapped (33, 151). The formation of DTP cells and the mechanism of drug resistance remain to be further investigated, and effective therapeutic targets remain to be discovered.

Currently, most DTP cells researches rely on *in vitro* cell experiments, and specific biomarkers to detect DTP cells in AML patients are still lacking in the process of clinical therapy. There is still a long way to go in applying tests in the laboratory to clinical practice. In the future, exploring the expression patterns of DTP cells in pathological tissues will be a key issue that will provide a basis for developing biomarkers for the early identification of DTP cells. With such biomarkers, DTP cells’ presence and dynamic changes can be monitored in real-time during clinical treatment, providing strong guidance for formulating personalized treatment plans.

The rise of high-throughput single-cell technology has opened up new avenues for DTP cell research (152). With single-cell sequencing technology, it is possible to precisely analyze the essential differences between ordinary cancer cells, DTP cells, and cancer stem cells in corresponding cancers (153, 154). This analysis helps to understand the origin and maintenance mechanisms of DTP cells deeply, reveal their roles at the microscopic level.

Promoting the translation of novel therapeutic strategies targeting DTP cells from the laboratory to clinical application is crucial for future research (46). Many promising anti-DTP drug candidates are still in the preclinical research stage, and their actual therapeutic effects in humans are unclear. However, some low-toxicity or non-toxic drugs and natural products may have inhibitory effects on DTP cells, such as discovering the anti-cancer potential of non-oncology drugs (33, 101, 151). Moreover, the combined use of multiple targeted drugs, such as epigenetic and metabolic inhibitors, may simultaneously block multiple drug resistance mechanisms of DTP cells, enhancing the therapeutic effect. These novel therapeutic strategies may lead to more efficient AML treatment and their clinical application feasibility should be further investigated.

However, although numerous studies of DTP cells have extensively outlined the details of resistance mechanisms, many directions of research into the DTP cells in AML remain to be pursued. Biomarkers for DTP cells should be discovered to provide them with accurate identification and monitoring in clinical treatment. High-throughput single-cell technology in research on DTP cells needs further development to understand their nature and provide high-precision analysis. Translating novel therapeutic strategies, equally essential, from concept into clinical practice must be accelerated. Only by advancing these research directions comprehensively can we overcome the drug resistance challenges in AML treatment, effectively improve patients’ therapeutic outcomes and survival rates, and bring a brighter future for AML patients.

## References

[1] Bray F Laversanne M Sung H Ferlay J Siegel R Soerjomataram I Global cancer statistics 2022: Globocan estimates of incidence and mortality worldwide for 36 cancers in 185 countries. *CA Cancer J Clin.* (2024) 74:229–63. 10.3322/caac.21834 38572751

[2] He S Xia C Li H Cao M Yang F Yan X Cancer profiles in China and comparisons with the USA: A comprehensive analysis in the incidence, mortality, survival, staging, and attribution to risk factors. *Sci China Life Sci.* (2024) 67:122–31. 10.1007/s11427-023-2423-1 37755589

[3] Kantarjian H Kadia T DiNardo C Daver N Borthakur G Jabbour E Acute myeloid leukemia: Current progress and future directions. *Blood Cancer J.* (2021) 11:41. 10.1038/s41408-021-00425-3 33619261 PMC7900255

[4] Papaemmanuil E Gerstung M Bullinger L Gaidzik V Paschka P Roberts N Genomic classification and prognosis in acute myeloid leukemia. *N Engl J Med.* (2016) 374:2209–21. 10.1056/NEJMoa1516192 27276561 PMC4979995

[5] Ley T Miller C Ding L Raphael B Mungall A Genomic and epigenomic landscapes of adult de novo acute myeloid leukemia. *N Engl J Med.* (2013) 368:2059–74. 10.1056/NEJMoa1301689 23634996 PMC3767041

[6] Tebbi C. Etiology of acute leukemia: A review. *Cancers (Basel).* (2021) 13:2256. 10.3390/cancers13092256 34066700 PMC8125807

[7] Miranda-Filho A Piñeros M Ferlay J Soerjomataram I Monnereau A Bray F. Epidemiological patterns of leukaemia in 184 countries: A population-based study. *Lancet Haematol.* (2018) 5:e14–24. 10.1016/S2352-3026(17)30232-6 29304322

[8] Bhansali R Pratz K Lai C. Recent advances in targeted therapies in acute myeloid leukemia. *J Hematol Oncol.* (2023) 16:29. 10.1186/s13045-023-01424-6 36966300 PMC10039574

[9] Short N Konopleva M Kadia T Borthakur G Ravandi F DiNardo C Advances in the treatment of acute myeloid leukemia: New drugs and new challenges. *Cancer Discov.* (2020) 10:506–25. 10.1158/2159-8290.CD-19-1011 32014868

[10] Ravandi F Cortes J Faderl S O’Brien S Garcia-Manero G Verstovsek S Characteristics and outcome of patients with acute myeloid leukemia refractory to 1 cycle of high-dose cytarabine-based induction chemotherapy. *Blood.* (2010) 116:5818–23. 10.1182/blood-2010-07-296392 20923968 PMC4081278

[11] Dinavahi S Gowda R Gowda K Bazewicz C Chirasani V Battu M Development of a novel multi-isoform ALDH inhibitor effective as an antimelanoma agent. *Mol Cancer Ther.* (2020) 19:447–59. 10.1158/1535-7163.MCT-19-0360 31754071 PMC10763724

[12] Kantarjian H Kadia T DiNardo C Welch M Ravandi F. Acute myeloid leukemia: Treatment and research outlook for 2021 and the MD Anderson approach. *Cancer.* (2021) 127:1186–207. 10.1002/cncr.33477 33734442 PMC12084862

[13] van Gils N Verhagen H Broux M Martiáñez T Denkers F Vermue E Targeting histone methylation to reprogram the transcriptional state that drives survival of drug-tolerant myeloid leukemia persisters. *iScience.* (2022) 25:105013. 10.1016/j.isci.2022.105013 36097617 PMC9463578

[14] Wang X Huang R Zhang X Zhang X. Current status and prospects of hematopoietic stem cell transplantation in China. *Chin Med J (Engl).* (2022) 135:1394–403. 10.1097/CM9.0000000000002235 35866344 PMC9481431

[15] Morgenstern Y Lee J Na Y Lieng B Ly N Gwynne W Acute myeloid leukemia drug-tolerant persister cells survive chemotherapy by transiently increasing plasma membrane rigidity, that also increases their sensitivity to immune cell killing. *Haematologica.* (2024) 110:893–903. 10.3324/haematol.2024.286018 39568440 PMC11962361

[16] Song X Lan Y Zheng X Zhu Q Liao X Liu K Targeting drug-tolerant cells: A promising strategy for overcoming acquired drug resistance in cancer cells. *MedComm.* (2023) 4:e342. 10.1002/mco2.342 37638338 PMC10449058

[17] Sharma S Lee D Li B Quinlan M Takahashi F Maheswaran S A chromatin-mediated reversible drug-tolerant state in cancer cell subpopulations. *Cell.* (2010) 141:69–80. 10.1016/j.cell.2010.02.027 20371346 PMC2851638

[18] Kaldalu N Tenson T. Slow growth causes bacterial persistence. *Sci Signal.* (2019) 12:eaay1167. 10.1126/scisignal.aay1167 31363066

[19] Balaban N Gerdes K Lewis K McKinney J. A problem of persistence: Still more questions than answers? *Nat Rev Microbiol.* (2013) 11:587–91. 10.1038/nrmicro3076 24020075

[20] Shen S Vagner S Robert C. Persistent cancer cells: The deadly survivors. *Cell.* (2020) 183:860–74. 10.1016/j.cell.2020.10.027 33186528

[21] Chang C Jen J Jiang S Sayad A Mer A Brown K Ontogeny and vulnerabilities of drug-tolerant persisters in HER2+ breast cancer. *Cancer Discov.* (2022) 12:1022–45. 10.1158/2159-8290.CD-20-1265 34911733 PMC8983469

[22] Ramirez M Rajaram S Steininger R Osipchuk D Roth M Morinishi L Diverse drug-resistance mechanisms can emerge from drug-tolerant cancer persister cells. *Nat Commun.* (2016) 7:10690. 10.1038/ncomms10690 26891683 PMC4762880

[23] Cabanos H Hata A. Emerging insights into targeted therapy-tolerant persister cells in cancer. *Cancers (Basel).* (2021) 13:2666. 10.3390/cancers13112666 34071428 PMC8198243

[24] De Conti G Dias M Bernards R. Fighting drug resistance through the targeting of drug-tolerant persister cells. *Cancers (Basel).* (2021) 13:1118. 10.3390/cancers13051118 33807785 PMC7961328

[25] Fahrer J. Switching off DNA repair-how colorectal cancer evades targeted therapies through adaptive mutability. *Signal Transduct Target Ther.* (2020) 5:19. 10.1038/s41392-020-0120-3 32296051 PMC7035417

[26] Wang X Zhang H Chen X. Drug resistance and combating drug resistance in cancer. *Cancer Drug Resist.* (2019) 2:141–60. 10.20517/cdr.2019.10 34322663 PMC8315569

[27] Karki P Angardi V Mier J Orman M. A transient metabolic state in melanoma persister cells mediated by chemotherapeutic treatments. *Front Mol Biosci.* (2022) 8:780192. 10.3389/fmolb.2021.780192 35155562 PMC8829428

[28] Izumi M Costa D Kobayashi S. Targeting of drug-tolerant persister cells as an approach to counter drug resistance in non-small cell lung cancer. *Lung Cancer.* (2024) 194:107885. 10.1016/j.lungcan.2024.107885 39002493 PMC11305904

[29] Ishida T Takahashi T Kurokawa Y Nishida T Hirota S Serada S Targeted therapy for drug-tolerant persister cells after imatinib treatment for gastrointestinal stromal tumours. *Br J Cancer.* (2021) 125:1511–22. 10.1038/s41416-021-01566-9 34611306 PMC8608810

[30] Szebényi K Füredi A Bajtai E Sama S Csiszar A Gombos B Effective targeting of breast cancer by the inhibition of P-glycoprotein mediated removal of toxic lipid peroxidation byproducts from drug tolerant persister cells. *Drug Resist Updat.* (2023) 71:101007. 10.1016/j.drup.2023.101007 37741091

[31] Böpple K Oren Y Henry W Dong M Weller S Thiel J ATF3 characterizes aggressive drug-tolerant persister cells in HGSOC. *Cell Death Dis.* (2024) 15:290. 10.1038/s41419-024-06674-x 38658567 PMC11043376

[32] Álvarez-Varela A Novellasdemunt L Barriga F Hernando-Momblona X Cañellas-Socias A Cano-Crespo S Mex3a marks drug-tolerant persister colorectal cancer cells that mediate relapse after chemotherapy. *Nat Cancer.* (2022) 3:1052–70. 10.1038/s43018-022-00402-0 35773527

[33] Gebru M Atkinson J Young M Zhang L Tang Z Liu Z Glucocorticoids enhance the antileukemic activity of FLT3 inhibitors in FLT3-mutant acute myeloid leukemia. *Blood.* (2020) 136:1067–79. 10.1182/blood.2019003124 32396937 PMC7453151

[34] Morgenstern Y Lee J Na Y Gwynne W Hurren R Ma L AML Drug tolerant persister (DTP) cells survive chemotherapy by transiently altering cellular lipidomics to increase plasma membrane rigidity, but also increases sensitivity to immune cell killing. *Blood.* (2023) 142:2806. 10.1182/blood-2023-178995 PMC11962361 39568440

[35] Aitken M Ravandi F Patel K Short N. Prognostic and therapeutic implications of measurable residual disease in acute myeloid leukemia. *J Hematol Oncol.* (2021) 14:137. 10.1186/s13045-021-01148-5 34479626 PMC8417965

[36] Verigou E Chatzilygeroudi T Lazaris V de Lastic A Symeonidis A. Immunophenotyping myelodysplastic neoplasms: The role of flow cytometry in the molecular classification era. *Front Oncol.* (2024) 14:1447001. 10.3389/fonc.2024.1447001 39544295 PMC11560873

[37] Raha D Wilson T Peng J Peterson D Yue P Evangelista M The cancer stem cell marker aldehyde dehydrogenase is required to maintain a drug-tolerant tumor cell subpopulation. *Cancer Res.* (2014) 74:3579–90. 10.1158/0008-5472.CAN-13-3456 24812274

[38] Moghal N Li Q Stewart E Navab R Mikubo M D’Arcangelo E Single-Cell analysis reveals transcriptomic features of drug-tolerant persisters and stromal adaptation in a patient-derived EGFR-mutated lung adenocarcinoma xenograft model. *J Thorac Oncol.* (2023) 18:499–515. 10.1016/j.jtho.2022.12.003 36535627

[39] Rehman S Haynes J Collignon E Brown K Wang Y Nixon A Colorectal cancer cells enter a diapause-like DTP state to survive chemotherapy. *Cell.* (2021) 184: 226–42.e21. 10.1016/j.cell.2020.11.018 33417860 PMC8437243

[40] Nojima Y Yao R Suzuki T. Single-cell RNA sequencing and machine learning provide candidate drugs against drug-tolerant persister cells in colorectal cancer. *Biochim Biophys Acta Mol Basis Dis.* (2025) 1871:167693. 10.1016/j.bbadis.2025.167693 39870146

[41] Haven B Heilig E Donham C Settles M Vasilevsky N Owen K Registered report: A chromatin-mediated reversible drug-tolerant state in cancer cell subpopulations. *Elife.* (2016) 5:e09462. 10.7554/eLife.09462 26905833 PMC4775209

[42] Kasbekar M Mitchell C Proven M Passegué E. Hematopoietic stem cells through the ages: A lifetime of adaptation to organismal demands. *Cell Stem Cell.* (2023) 30:1403–20. 10.1016/j.stem.2023.09.013 37865087 PMC10842631

[43] Stelmach P Trumpp A. Leukemic stem cells and therapy resistance in acute myeloid leukemia. *Haematologica.* (2023) 108:353–66. 10.3324/haematol.2022.280800 36722405 PMC9890038

[44] Eaves C. Hematopoietic stem cells: Concepts, definitions, and the new reality. *Blood.* (2015) 125:2605–13. 10.1182/blood-2014-12-570200 25762175 PMC4440889

[45] Buss E Ho A. Leukemia stem cells. *Int J Cancer.* (2011) 129:2328–36. 10.1002/ijc.26318 21796620

[46] Russo M Chen M Mariella E Peng H Rehman S Sancho E Cancer drug-tolerant persister cells: From biological questions to clinical opportunities. *Nat Rev Cancer.* (2024) 24:694–717. 10.1038/s41568-024-00737-z 39223250 PMC12622869

[47] Varisli L Dancik G Copland J Vlahopoulos S. Editorial: Acute leukemias: molecular characterization, leukemia-initiating cells, and influence of the microenvironment, volume II. *Front Oncol.* (2025) 14:1542306. 10.3389/fonc.2024.1542306 39834938 PMC11743270

[48] Herrmann H Sadovnik I Eisenwort G Rülicke T Blatt K Herndlhofer S Delineation of target expression profiles in CD34+/CD38- and CD34+/CD38+ stem and progenitor cells in AML and CML. *Blood Adv.* (2020) 4:5118–32. 10.1182/bloodadvances.2020001742 33085758 PMC7594398

[49] Bell C Gilan O. Principles and mechanisms of non-genetic resistance in cancer. *Br J Cancer.* (2020) 122:465–72. 10.1038/s41416-019-0648-6 31831859 PMC7028722

[50] Duy C Li M Teater M Meydan C Garrett-Bakelman F Lee T Chemotherapy induces senescence-like resilient cells capable of initiating AML recurrence. *Cancer Discov.* (2021) 11:1542–61. 10.1158/2159-8290.CD-20-1375 33500244 PMC8178167

[51] Dhimolea E de Matos Simoes R Kansara D Al’Khafaji A Bouyssou J Weng X An embryonic diapause-like adaptation with suppressed myc activity enables tumor treatment persistence. *Cancer Cell.* (2021) 39: 240–56.e11. 10.1016/j.ccell.2020.12.002 33417832 PMC8670073

[52] Bulut-Karslioglu A Biechele S Jin H Macrae T Hejna M Gertsenstein M Inhibition of mTOR induces a paused pluripotent state. *Nature.* (2016) 540:119–23. 10.1038/nature20578 27880763 PMC5143278

[53] Jones C Stevens B Pollyea D Culp-Hill R Reisz J Nemkov T Nicotinamide metabolism mediates resistance to venetoclax in relapsed acute myeloid leukemia stem cells. *Cell Stem Cell.* (2020) 27:748–64.e4. 10.1016/j.stem.2020.07.021 32822582 PMC7655603

[54] Li Z Guo Z Xiao H Chen X Liu W Zhou H. Simulating neuronal development: Exploring potential mechanisms for central nervous system metastasis in acute lymphoblastic leukemia. *Front Oncol.* (2023) 13:1331802. 10.3389/fonc.2023.1331802 38239636 PMC10794646

[55] Goddard E Linde M Srivastava S Klug G Shabaneh T Iannone S Immune evasion of dormant disseminated tumor cells is due to their scarcity and can be overcome by T cell immunotherapies. *Cancer Cell.* (2024) 42: 119–34.e12. 10.1016/j.ccell.2023.12.011 38194912 PMC10864018

[56] Fu D Zhang B Wu S Feng J Jiang H. Molecular subtyping of acute myeloid leukemia through ferroptosis signatures predicts prognosis and deciphers the immune microenvironment. *Front Cell Dev Biol.* (2023) 11:1207642. 10.3389/fcell.2023.1207642 37691822 PMC10483833

[57] van Weverwijk A de Visser K. Mechanisms driving the immunoregulatory function of cancer cells. *Nat Rev Cancer.* (2023) 23:193–215. 10.1038/s41568-022-00544-4 36717668

[58] Zhang Z Qin S Chen Y Zhou L Yang M Tang Y Inhibition of NPC1L1 disrupts adaptive responses of drug-tolerant persister cells to chemotherapy. *EMBO Mol Med.* (2022) 14:e14903. 10.15252/emmm.202114903 35023619 PMC8819355

[59] Wang Y Wang Q Ren H Dong Y Wang Q Liang Z Efficacy and safety of hypomethylating agents in the treatment of AML/MDS patients relapsed post allogenetic hematopoietic stem cell transplantation. *Front Oncol.* (2024) 14:1465334. 10.3389/fonc.2024.1465334 39717745 PMC11663890

[60] Patel S Nilsson M Yang Y Shen L Wang J Poteete A Targeting IL-6/STAT3 signaling in EGFR-mutant drug tolerant persister cells. *Cancer Res.* (2023) 83:3864. 10.1158/1538-7445.AM2023-3864

[61] Shih P Mei K. Role of STAT3 signaling transduction pathways in cancer stem cell-associated chemoresistance. *Drug Discov Today.* (2021) 26:1450–8. 10.1016/j.drudis.2020.11.032 33307211

[62] Bourguignon L Peyrollier K Xia W Gilad E. Hyaluronan-CD44 interaction activates stem cell marker Nanog, Stat-3-mediated MDR1 gene expression, and ankyrin-regulated multidrug efflux in breast and ovarian tumor cells. *J Biol Chem.* (2008) 283:17635–51. 10.1074/jbc.M800109200 18441325 PMC2427357

[63] Guo Q Yang C Gao F. The state of CD44 activation in cancer progression and therapeutic targeting. *FEBS J.* (2022) 289:7970–86. 10.1111/febs.16179 34478583

[64] Radhakrishnan A Shanmukhan N Samuel L. Pharmacogenomics influence on MDR1-associated cancer resistance and innovative drug delivery approaches: Advancing precision oncology. *Med Oncol.* (2025) 42:67. 10.1007/s12032-025-02611-w 39913003

[65] Pelletier J Graff J Ruggero D Sonenberg N. Targeting the eIF4F translation initiation complex: A critical nexus for cancer development. *Cancer Res.* (2015) 75:250–63. 10.1158/0008-5472.CAN-14-2789 25593033 PMC4299928

[66] Shen S Faouzi S Bastide A Martineau S Malka-Mahieu H Fu Y An epitranscriptomic mechanism underlies selective mRNA translation remodelling in melanoma persister cells. *Nat Commun.* (2019) 10:5713. 10.1038/s41467-019-13360-6 31844050 PMC6915789

[67] Bordeleau M Mori A Oberer M Lindqvist L Chard L Higa T Functional characterization of IRESes by an inhibitor of the RNA helicase eIF4A. *Nat Chem Biol.* (2006) 2:213–20. 10.1038/nchembio776 16532013

[68] Zhang Z Tan Y Huang C Wei X. Redox signaling in drug-tolerant persister cells as an emerging therapeutic target. *EBioMedicine.* (2023) 89:104483. 10.1016/j.ebiom.2023.104483 36827719 PMC9982619

[69] Li Y Chen H Xie X Yang B Wang X Zhang J PINK1-mediated mitophagy promotes oxidative phosphorylation and redox homeostasis to induce drug-tolerant persister cancer cells. *Cancer Res.* (2023) 83:398–413. 10.1158/0008-5472.CAN-22-2370 36480196

[70] Tjahjono E Daneman M Meika B Revtovich A Kirienko N. Mitochondrial abnormalities as a target of intervention in acute myeloid leukemia. *Front Oncol.* (2025) 14:1532857. 10.3389/fonc.2024.1532857 39902131 PMC11788353

[71] Farge T Saland E de Toni F Aroua N Hosseini M Perry R Chemotherapy-resistant human acute myeloid leukemia cells are not enriched for leukemic stem cells but require oxidative metabolism. *Cancer Discov.* (2017) 7:716–35. 10.1158/2159-8290.CD-16-0441 28416471 PMC5501738

[72] Zhang X Ma Y Ma J Yang L Song Q Wang H Glutathione peroxidase 4 as a therapeutic target for anti-colorectal cancer drug-tolerant persister cells. *Front Oncol.* (2022) 12:913669. 10.3389/fonc.2022.913669 35719967 PMC9203854

[73] Wang S Wang Q Lv S Qin L. Prognostic value of serum lipids in newly diagnosed acute promyelocytic leukemia. *Front Oncol.* (2025) 15:1522239. 10.3389/fonc.2025.1522239 40040719 PMC11876187

[74] Nie M Hu Z. Metabolic orchestration of drug-tolerant persister cells in cancer. *Life Med.* (2024) 3:lnae040. 10.1093/lifemedi/lnae040 39872154 PMC11748267

[75] Dong C Wu J Chen Y Nie J Chen C. Activation of PI3K/AKT/mTOR pathway causes drug resistance in breast cancer. *Front Pharmacol.* (2021) 12:628690. 10.3389/fphar.2021.628690 33790792 PMC8005514

[76] Zhou H Liu W Zhou Y Hong Z Ni J Zhang X Therapeutic inhibition of GAS6-AS1/YBX1/MYC axis suppresses cell propagation and disease progression of acute myeloid leukemia. *J Exp Clin Cancer Res.* (2021) 40:353. 10.1186/s13046-021-02145-9 34753494 PMC8576903

[77] Tao L Huang G Song H Chen Y Chen L. Cancer associated fibroblasts: An essential role in the tumor microenvironment. *Oncol Lett.* (2017) 14:2611–20. 10.3892/ol.2017.6497 28927027 PMC5588104

[78] Tabe Y Konopleva M. Role of microenvironment in resistance to therapy in AML. *Curr Hematol Malig Rep.* (2015) 10:96–103. 10.1007/s11899-015-0253-6 25921386 PMC4447522

[79] Ayala F Dewar R Kieran M Kalluri R. Contribution of bone microenvironment to leukemogenesis and leukemia progression. *Leukemia.* (2009) 23:2233–41. 10.1038/leu.2009.175 19727127 PMC4313556

[80] Shafat M Gnaneswaran B Bowles K Rushworth S. The bone marrow microenvironment - Home of the leukemic blasts. *Blood Rev.* (2017) 31:277–86. 10.1016/j.blre.2017.03.004 28318761

[81] Ding Z Shi R Hu W Tian L Sun R Wu Y Cancer-associated fibroblasts in hematologic malignancies: Elucidating roles and spotlighting therapeutic targets. *Front Oncol.* (2023) 13:1193978. 10.3389/fonc.2023.1193978 37746306 PMC10511871

[82] Duan C Shi J Chen J Wang B Yu Y Qin X Leukemia propagating cells rebuild an evolving niche in response to therapy. *Cancer Cell.* (2014) 25:778–93. 10.1016/j.ccr.2014.04.015 24937459

[83] Burt R Dey A Aref S Aguiar M Akarca A Bailey K Activated stromal cells transfer mitochondria to rescue acute lymphoblastic leukemia cells from oxidative stress. *Blood.* (2019) 134:1415–29. 10.1182/blood.2019001398 31501154 PMC6856969

[84] Camacho V McClearn V Patel S Welner R. Regulation of normal and leukemic stem cells through cytokine signaling and the microenvironment. *Int J Hematol.* (2017) 105:566–77. 10.1007/s12185-017-2184-6 28176225

[85] Blonska M Agarwal N Vega F. Shaping of the tumor microenvironment: Stromal cells and vessels. *Semin Cancer Biol.* (2015) 34:3–13. 10.1016/j.semcancer.2015.03.002 25794825 PMC6374506

[86] Szondy Z Sarang Z Kiss B Garabuczi É Köröskényi K. Anti-inflammatory mechanisms triggered by apoptotic cells during their clearance. *Front Immunol.* (2017) 8:909. 10.3389/fimmu.2017.00909 28824635 PMC5539239

[87] Maxwell P Pugh C Ratcliffe P. Activation of the HIF pathway in cancer. *Curr Opin Genet Dev.* (2001) 11:293–9. 10.1016/s0959-437x(00)00193-3 11377966

[88] Aloia A Müllhaupt D Chabbert C Eberhart T Flückiger-Mangual S Vukolic A A fatty acid oxidation-dependent metabolic shift regulates the adaptation of BRAF-mutated melanoma to MAPK inhibitors. *Clin Cancer Res.* (2019) 25:6852–67. 10.1158/1078-0432.CCR-19-0253 31375515 PMC6906212

[89] Li S Song Y Quach C Guo H Jang G Maazi H Transcriptional regulation of autophagy-lysosomal function in BRAF-driven melanoma progression and chemoresistance. *Nat Commun.* (2019) 10:1693. 10.1038/s41467-019-09634-8 30979895 PMC6461621

[90] Gu L Liao P Liu H. Cancer-associated fibroblasts in acute leukemia. *Front Oncol.* (2022) 12:1022979. 10.3389/fonc.2022.1022979 36601484 PMC9806275

[91] Zhao Y Bai Y Shen M Li Y. Therapeutic strategies for gastric cancer targeting immune cells: Future directions. *Front Immunol.* (2022) 13:992762. 10.3389/fimmu.2022.992762 36225938 PMC9549957

[92] Liu S Li M Liang B Sun W Shao Y Hu X Breaking the barrier: Nanoparticle-enhanced radiotherapy as the new vanguard in brain tumor treatment. *Front Pharmacol.* (2024) 15:1394816. 10.3389/fphar.2024.1394816 39021831 PMC11252536

[93] Zhang H Dai Z Wu W Wang Z Zhang N Zhang L Regulatory mechanisms of immune checkpoints PD-L1 and CTLA-4 in cancer. *J Exp Clin Cancer Res.* (2021) 40:184. 10.1186/s13046-021-01987-7 34088360 PMC8178863

[94] Sun C Mezzadra R Schumacher T. Regulation and function of the PD-L1 checkpoint. *Immunity.* (2018) 48:434–52. 10.1016/j.immuni.2018.03.014 29562194 PMC7116507

[95] Ciszak L Frydecka I Wolowiec D Szteblich A Kosmaczewska A. Patients with chronic lymphocytic leukaemia (CLL) differ in the pattern of CTLA-4 expression on CLL cells: The possible implications for immunotherapy with CTLA-4 blocking antibody. *Tumour Biol.* (2016) 37:4143–57. 10.1007/s13277-015-4217-1 26490985 PMC4844645

[96] Swatler J Turos-Korgul L Kozlowska E Piwocka K. Immunosuppressive cell subsets and factors in myeloid leukemias. *Cancers (Basel).* (2021) 13:1203. 10.3390/cancers13061203 33801964 PMC7998753

[97] Nasef A Mazurier C Bouchet S François S Chapel A Thierry D Leukemia inhibitory factor: Role in human mesenchymal stem cells mediated immunosuppression. *Cell Immunol.* (2008) 253:16–22. 10.1016/j.cellimm.2008.06.002 18639869

[98] Wu H Li P Shao N Ma J Ji M Sun X Aberrant expression of Treg-associated cytokine IL-35 along with IL-10 and TGF-β in acute myeloid leukemia. *Oncol Lett.* (2012) 3:1119–23. 10.3892/ol.2012.614 22783403 PMC3389635

[99] Liu C Yin Q Wu Z Li W Huang J Chen B Inflammation and immune escape in ovarian cancer: Pathways and therapeutic opportunities. *J Inflamm Res.* (2025) 18:895–909. 10.2147/JIR.S503479 39867950 PMC11762012

[100] Haist M Stege H Grabbe S Bros M. The functional crosstalk between myeloid-derived suppressor cells and regulatory T cells within the immunosuppressive tumor microenvironment. *Cancers (Basel).* (2021) 13:210. 10.3390/cancers13020210 33430105 PMC7827203

[101] He J Qiu Z Fan J Xie X Sheng Q Sui X. Drug tolerant persister cell plasticity in cancer: A revolutionary strategy for more effective anticancer therapies. *Signal Transduct Target Ther.* (2024) 9:209. 10.1038/s41392-024-01891-4 39138145 PMC11322379

[102] Huang Y Chen C Sundaramurthy V Słabicki M Hao D Watson C Systematic profiling of DNMT3A variants reveals protein instability mediated by the DCAF8 E3 ubiquitin ligase adaptor. *Cancer Discov.* (2022) 12:220–35. 10.1158/2159-8290.CD-21-0560 34429321 PMC8758508

[103] Maemondo M Inoue A Kobayashi K Sugawara S Oizumi S Isobe H Gefitinib or chemotherapy for non-small-cell lung cancer with mutated EGFR. *N Engl J Med.* (2010) 362:2380–8. 10.1056/NEJMoa0909530 20573926

[104] Deng S Wang C Wang Y Xu Y Li X Johnson N Ectopic JAK-STAT activation enables the transition to a stem-like and multilineage state conferring AR-targeted therapy resistance. *Nat Cancer.* (2022) 3:1071–87. 10.1038/s43018-022-00431-9 36065066 PMC9499870

[105] Levis M. FLT3 dancing on the stem cell. *J Exp Med.* (2017) 214:1857–9. 10.1084/jem.20171056 28637882 PMC5502439

[106] Figueroa M Abdel-Wahab O Lu C Ward P Patel J Shih A Leukemic IDH1 and IDH2 mutations result in a hypermethylation phenotype, disrupt TET2 function, and impair hematopoietic differentiation. *Cancer Cell.* (2010) 18:553–67. 10.1016/j.ccr.2010.11.015 21130701 PMC4105845

[107] Chen SC Liao TT Yang MH. Emerging roles of epithelial-mesenchymal transition in hematological malignancies. *J Biomed Sci.* (2018). 25:37. 10.1186/s12929-018-0440-6 29685144 PMC5913878

[108] Suda K Tomizawa K Fujii M Murakami H Osada H Maehara Y Epithelial to mesenchymal transition in an epidermal growth factor receptor-mutant lung cancer cell line with acquired resistance to erlotinib. *J Thorac Oncol.* (2011) 6:1152–61. 10.1097/JTO.0b013e318216ee52 21597390

[109] Ishikawa F Yoshida S Saito Y Hijikata A Kitamura H Tanaka S Chemotherapy-resistant human AML stem cells home to and engraft within the bone-marrow endosteal region. *Nat Biotechnol.* (2007) 25:1315–21. 10.1038/nbt1350 17952057

[110] Möhle R Bautz F Rafii S Moore M Brugger W Kanz L. The chemokine receptor CXCR-4 is expressed on CD34+ hematopoietic progenitors and leukemic cells and mediates transendothelial migration induced by stromal cell-derived factor-1. *Blood.* (1998) 91:4523–30. 10.1182/blood.V91.12.4523 9616148

[111] Lagadinou E Sach A Callahan K Rossi R Neering S Minhajuddin M BCL-2 inhibition targets oxidative phosphorylation and selectively eradicates quiescent human leukemia stem cells. *Cell Stem Cell.* (2013) 12:329–41. 10.1016/j.stem.2012.12.013 23333149 PMC3595363

[112] Culp-Hill R D’Alessandro A Pietras E. Extinguishing the embers: Targeting AML metabolism. *Trends Mol Med.* (2021) 27:332–44. 10.1016/j.molmed.2020.10.001 33121874 PMC8005405

[113] Kobayashi C Suda T. Regulation of reactive oxygen species in stem cells and cancer stem cells. *J Cell Physiol.* (2012) 227:421–30. 10.1002/jcp.22764 21448925

[114] Kawakami R Mashima T Kawata N Kumagai K Migita T Sano T ALDH1A3-mTOR axis as a therapeutic target for anticancer drug-tolerant persister cells in gastric cancer. *Cancer Sci.* (2020) 111:962–73. 10.1111/cas.14316 31960523 PMC7060474

[115] Parmar K Mauch P Vergilio J Sackstein R Down J. Distribution of hematopoietic stem cells in the bone marrow according to regional hypoxia. *Proc Natl Acad Sci U S A.* (2007) 104:5431–6. 10.1073/pnas.0701152104 17374716 PMC1838452

[116] Mohyeldin A Garzón-Muvdi T Quiñones-Hinojosa A. Oxygen in stem cell biology: A critical component of the stem cell niche. *Cell Stem Cell.* (2010) 7:150–61. 10.1016/j.stem.2010.07.007 20682444

[117] Tothova Z Gilliland D. FoxO transcription factors and stem cell homeostasis: Insights from the hematopoietic system. *Cell Stem Cell.* (2007) 1:140–52. 10.1016/j.stem.2007.07.017 18371346

[118] Pei S Minhajuddin M Adane B Khan N Stevens B Mack S AMPK/FIS1-mediated mitophagy is required for self-renewal of human AML stem cells. *Cell Stem Cell.* (2018) 23: 86–100.e6. 10.1016/j.stem.2018.05.021 29910151 PMC6035102

[119] Wu Y Huang C Hsieh M Huang C Setiawan S Yeh C Targeting of FSP1 regulates iron homeostasis in drug-tolerant persister head and neck cancer cells via lipid-metabolism-driven ferroptosis. *Aging (Albany NY).* (2024) 16:627–47. 10.18632/aging.205409 38206305 PMC10817390

[120] Sui X Zhang R Liu S Duan T Zhai L Zhang M RSL3 drives ferroptosis through GPX4 inactivation and ROS production in colorectal cancer. *Front Pharmacol.* (2018) 9:1371. 10.3389/fphar.2018.01371 30524291 PMC6262051

[121] Pu Y Li L Peng H Liu L Heymann D Robert C Drug-tolerant persister cells in cancer: The cutting edges and future directions. *Nat Rev Clin Oncol.* (2023) 20:799–813. 10.1038/s41571-023-00815-5 37749382

[122] Yin Q Wang M Hu A Gao J Llaneza D Blackburn L Combinatorial therapy of CDK9 inhibitor with CD19 CAR-T to reciprocally overcome therapy resistance and enhance treatment efficacies against aggressive B-cell lymphomas. *Blood.* (2024) 144:6219. 10.1182/blood-2024-204209

[123] Li S Siriwon N Zhang X Yang S Jin T He F Enhanced cancer immunotherapy by chimeric antigen receptor-modified T cells engineered to secrete checkpoint inhibitors. *Clin Cancer Res.* (2017) 23:6982–92. 10.1158/1078-0432.CCR-17-0867 28912137

[124] Rafiq S Yeku O Jackson H Purdon T van Leeuwen D Drakes D Targeted delivery of a PD-1-blocking scFv by CAR-T cells enhances anti-tumor efficacy in vivo. *Nat Biotechnol.* (2018) 36:847–56. 10.1038/nbt.4195 30102295 PMC6126939

[125] Wang X Zhang Y Xue S. Recent progress in chimeric antigen receptor therapy for acute myeloid leukemia. *Ann Hematol.* (2024) 103:1843–57. 10.1007/s00277-023-05601-y 38381173

[126] Lichtenegger F Krupka C Haubner S Köhnke T Subklewe M. Recent developments in immunotherapy of acute myeloid leukemia. *J Hematol Oncol.* (2017) 10:1–20. 10.1186/s13045-017-0505-0 28743264 PMC5526264

[127] Banelli B Carra E Barbieri F Würth R Parodi F Pattarozzi A The histone demethylase KDM5A is a key factor for the resistance to temozolomide in glioblastoma. *Cell Cycle.* (2015) 14:3418–29. 10.1080/15384101.2015.1090063 26566863 PMC4825557

[128] Emran A Marzese D Menon D Stark M Torrano J Hammerlindl H Distinct histone modifications denote early stress-induced drug tolerance in cancer. *Oncotarget.* (2018) 9:8206–22. 10.18632/oncotarget.23654 29492189 PMC5823586

[129] Sarno F Nebbioso A Altucci L. *Histone Demethylase Inhibitors and Their Potential in Cancer Treatment. Histone Modifications in Therapy.* Amsterdam: Elsevier (2020). p. 143–77.

[130] Zhang X Li L Li Y Dong C Shi J Guo X The role of trimethylation on histone H3 lysine 27 (H3K27me3) in temozolomide resistance of glioma. *Brain Res.* (2025) 1846:149252. 10.1016/j.brainres.2024.149252 39326722

[131] Li Y Zhang M Sheng M Zhang P Chen Z Xing W Therapeutic potential of GSK-J4, a histone demethylase KDM6B/JMJD3 inhibitor, for acute myeloid leukemia. *J Cancer Res Clin Oncol.* (2018) 144:1065–77. 10.1007/s00432-018-2631-7 29594337 PMC5948279

[132] Marin-Bejar O Rogiers A Dewaele M Femel J Karras P Pozniak J Evolutionary predictability of genetic versus nongenetic resistance to anticancer drugs in melanoma. *Cancer Cell.* (2021) 39: 1135–49.e8. 10.1016/j.ccell.2021.05.015 34143978

[133] Pham V Pitti R Tindell C Cheung T Masselot A Stephan J Proteomic analyses identify a novel role for EZH2 in the initiation of cancer cell drug tolerance. *J Proteome Res.* (2020) 19:1533–47. 10.1021/acs.jproteome.9b00773 32159963

[134] Chen Y Wang J Zhang F Liu P. A perspective of immunotherapy for acute myeloid leukemia: Current advances and challenges. *Front Pharmacol.* (2023) 14:1151032. 10.3389/fphar.2023.1151032 37153761 PMC10154606

[135] Feins S Kong W Williams E Milone M Fraietta J. An introduction to chimeric antigen receptor (CAR) T-cell immunotherapy for human cancer. *Am J Hematol.* (2019) 94:S3–9. 10.1002/ajh.25418 30680780

[136] June C O’Connor R Kawalekar O Ghassemi S Milone MC. CAR T cell immunotherapy for human cancer. *Science.* (2018) 359:1361–5. 10.1126/science.aar6711 29567707

[137] Vishwasrao P Li G Boucher J Smith D Hui S. Emerging CAR T cell strategies for the treatment of AML. *Cancers (Basel).* (2022) 14:1241. 10.3390/cancers14051241 35267549 PMC8909045

[138] Alcantara M Du Rusquec P Romano E. Current clinical evidence and potential solutions to increase benefit of CAR T-cell therapy for patients with solid tumors. *Oncoimmunology.* (2020) 9:1777064. 10.1080/2162402X.2020.1777064 32934880 PMC7466853

[139] Shah N Fry T. Mechanisms of resistance to CAR T cell therapy. *Nat Rev Clin Oncol.* (2019) 16:372–85. 10.1038/s41571-019-0184-6 30837712 PMC8214555

[140] Rotte A. Combination of CTLA-4 and PD-1 blockers for treatment of cancer. *J Exp Clin Cancer Res.* (2019) 38:255. 10.1186/s13046-019-1259-z 31196207 PMC6567914

[141] Buchbinder E Desai A. CTLA-4 and PD-1 pathways: Similarities, differences, and implications of their inhibition. *Am J Clin Oncol.* (2016) 39:98–106. 10.1097/COC.0000000000000239 26558876 PMC4892769

[142] Wojtukiewicz M Rek M Karpowicz K Górska M Polityńska B Wojtukiewicz A Inhibitors of immune checkpoints-PD-1, PD-L1, CTLA-4-new opportunities for cancer patients and a new challenge for internists and general practitioners. *Cancer Metastasis Rev.* (2021) 40:949–82. 10.1007/s10555-021-09976-0 34236546 PMC8556173

[143] Bawek S Gurusinghe S Burwinkel M Przespolewski A. Updates in novel immunotherapeutic strategies for relapsed/refractory AML. *Front Oncol.* (2024) 14:1374963. 10.3389/fonc.2024.1374963 39697225 PMC11652486

[144] McDonald P Dedhar S. Persister cell plasticity in tumour drug resistance. *Semin Cell Dev Biol.* (2024) 156:1–10. 10.1016/j.semcdb.2023.11.003 37977107

[145] Zhang C Liang S Zhang H Wang R Qiao H. Epigenetic regulation of mRNA mediates the phenotypic plasticity of cancer cells during metastasis and therapeutic resistance (Review). *Oncol Rep.* (2023) 51:28. 10.3892/or.2023.8687 38131215 PMC10777459

[146] Li Y Chen H Lu D Koeffler H Zhang Y Yin D. Mitophagy is a novel protective mechanism for drug-tolerant persister (DTP) cancer cells. *Autophagy.* (2023) 19:2618–9. 10.1080/15548627.2023.2177398 36747349 PMC10392730

[147] Mani N Daiya A Chowdhury R Mukherjee S Chowdhury S. Epigenetic adaptations in drug-tolerant tumor cells. *Adv Cancer Res.* (2023) 158:293–335. 10.1016/bs.acr.2022.12.006 36990535

[148] Perez-Medina M Lopez-Gonzalez J Benito-Lopez J Ávila-Ríos S Soto-Nava M Matias-Florentino M Transcriptomic analysis reveals early alterations associated with intrinsic resistance to targeted therapy in lung adenocarcinoma cell lines. *Cancers (Basel).* (2024) 16:2490. 10.3390/cancers16132490 39001552 PMC11240825

[149] Kunimasa K Nagano T Shimono Y Dokuni R Kiriu T Tokunaga S Glucose metabolism-targeted therapy and withaferin A are effective for epidermal growth factor receptor tyrosine kinase inhibitor-induced drug-tolerant persisters. *Cancer Sci.* (2017) 108:1368–77. 10.1111/cas.13266 28445002 PMC5497794

[150] Dhanyamraju P Schell T Amin S Robertson G. Drug-tolerant persister cells in cancer therapy resistance. *Cancer Res*. (2022) 82:2503–14. 10.1158/0008-5472.CAN-21-3844 35584245 PMC9296591

[151] Corsello S Nagari R Spangler R Rossen J Kocak M Bryan J Discovering the anti-cancer potential of non-oncology drugs by systematic viability profiling. *Nat Cancer.* (2020) 1:235–48. 10.1038/s43018-019-0018-6 32613204 PMC7328899

[152] Hare P LaGree T Byrd B DeMarco A Mok W. Single-cell technologies to study phenotypic heterogeneity and bacterial persisters. *Microorganisms.* (2021) 9:2277. 10.3390/microorganisms9112277 34835403 PMC8620850

[153] van Galen P Hovestadt V. Wadsworth Ii MH Hughes TK Griffin GK Battaglia S Single-*Cell* RNA-Seq reveals AML hierarchies relevant to disease progression and immunity. *Cell.* (2019) 176:1265–81.e24. 10.1016/j.cell.2019.01.031 30827681 PMC6515904

[154] Ediriwickrema A Gentles A Majeti R. Single-cell genomics in AML: Extending the frontiers of AML research. *Blood.* (2023) 14:345–55. 10.1182/blood.2021014670 35926108 PMC10082362

